# Relationship between Cancer and Intention to Leave Work among Older Workers: A Cross-Sectional Internet-Based Study

**DOI:** 10.3390/medicina60091506

**Published:** 2024-09-15

**Authors:** Ryutaro Matsugaki, Shinya Matsuda, Akira Ogami

**Affiliations:** 1 Department of Work Systems and Health, Institute of Industrial Ecological Sciences, University of Occupational and Environmental Health, Kitakyushu 807-8555, Japan; 2 Department of Preventive Medicine and Community Health, University of Occupational and Environmental Health, Kitakyushu 807-8555, Japan

**Keywords:** cancer, intention to leave, older worker, unemployment

## Abstract

*Background and Objectives*: Limited research has focused on the relationship between cancer, job loss, and factors associated with job loss among older workers. Therefore, in this study, we aimed to examine the relationship between cancer and intention to leave and between physical-health-related issues, mental-health-related issues, and cancer-related symptoms and intention to leave among older workers with cancer. *Materials and Methods*: This cross-sectional internet-based study included 4498 workers aged 60–75 years. Intention to leave was assessed based on whether individuals considered quitting their current jobs in the near future. *Results*: A multivariate logistic regression analysis showed a significant association between cancer and intention to leave (adjusted odds ratio [aOR]: 1.42, 95% confidence interval [CI]: 1.01–2.00, *p* = 0.045). In addition, physical-health-related issues (aOR: 2.33, 95% CI: 1.10–4.92, *p* = 0.026) and mental-health-related issues (aOR: 4.44, 95% CI: 1.80–10.98, *p* = 0.001) were significantly associated with the intention to leave. *Conclusions:* Healthcare providers and employers must address the physical- and mental-health-related issues facing older workers with cancer to help them secure their employment.

## 1. Introduction

The diagnosis and treatment of cancer significantly impact individuals’ lives. Recent advances in medicine, especially in high-income countries, have improved the survival rates of patients with cancer [1,2,3], highlighting the importance of research on the social and occupational challenges faced by survivors of cancer. Unemployment is a critical issue among survivors of cancer. Survivors of cancer have a higher rate of job loss than the general population [4,5,6], and unemployment can lead to financial toxicity [7,8,9] and a reduction in quality of life [10,11,12,13,14]. Therefore, the demand for strategies to avoid unemployment among patients with cancer is increasing.

Notably, several factors have been associated with unemployment among survivors of cancer. Personal factors include older age [6,14,15,16,17], female sex [6,18], low educational level [6,19], lower income level [18,19], physically or mentally demanding work [17,19], clinical stage [15,16,19], and type of treatment [15,16,17,19]. The modifiable factors include physical-health-related issues such as low fitness levels and poor physical function [17], mental-health-related issues such as depression [20], and cancer-related symptoms such as fatigue and pain [21,22,23,24,25]. Therefore, healthcare providers, employers, and occupational health staff should address these physical- and mental-health-related issues and cancer-related symptoms to prevent job loss among patients with cancer.

The incidence of cancer is closely associated with aging, peaking in the population in their 60s and 70s [26,27]. Furthermore, the rise in the aging population and retirement-age groups in developed countries has led to a corresponding increase in the proportion of older workers in the labor market [28]. At the intersection of these trends, the employment challenges faced by older survivors of cancer are expected to become increasingly significant. However, limited research has focused on the relationship between cancer, job loss, and factors associated with job loss among older workers. In the present study, we aimed to (1) clarify whether cancer is associated with intentions to leave work among older workers and (2) identify whether physical- and mental-health-related issues and cancer-related symptoms are associated with intentions to leave work among workers with cancer.

## 2. Materials and Methods

### 2.1. Study Design and Participants

This cross-sectional internet-based study was conducted to explore the employment and health challenges faced by older workers [29]. The survey targeted workers aged 60–75 years who were employed in the tertiary sector and registered with Cross Marketing Inc. (Tokyo, Japan).

On 1 September 2022, invitations were sent via email or other methods to those panelists who, at the time of their registration, indicated they were employed. The criteria for exclusion included (1) unemployment at the time of the survey, (2) being younger than 60 years or older than 75 years, (3) being self-employed or a family member actively participating in a family business, (4) not working in the tertiary sector as defined by the Indices of Tertiary Industry Activity in Japan, and (5) incorrect answers to a control question designed to filter out unreliable data: “Identify the third largest number from a list of five numbers”. The recruitment process concluded on 9 September 2022, with the target of 5000 participants met. Fifty-two individuals who anticipated imminent retirement due to reasons such as reaching retirement age or contract termination for the purposes of this research were excluded, resulting in a final sample size of 4948 participants. All the participants provided informed consent online through a survey interface. The study protocol was approved by the Ethics Committee of the University of Occupational and Environmental Health, Japan (R4-031).

### 2.2. Assessment of the Prevalence of Cancer

All participants were asked the question, “What illnesses do you have that require outpatient visits or treatment?”. Those who responded “yes” to the option for cancer were considered diagnosed with cancer.

### 2.3. Assessment of Physical, Mental, and Symptomatic Health-Related Issues

In this study, we investigated the health-related problems faced by the participants that prevented them from continuing their current employment. Notably, we focused on physical- and mental-health-related issues and cancer-related symptoms. The participants were asked the question, “What kind of problems are you encountering in order to continue your current job?”. Subsequently, the participants were presented with three items: “physical fitness-related problems”, “mental health-related problems”, and “problems associated with cancer-related symptoms”. For each item, the participants were required to respond with either “Yes” or “No”. This approach allowed for ascertaining the various health-related challenges encountered by survivors of cancer in their workplace and quantitatively assessing these barriers to sustained employment.

### 2.4. Assessment of Intention to Leave Work

We assessed the participants’ intention to leave using the question “Do you intend to leave your current job in the near future?” with the following response options: “Sure”, “I think so”, or “Definitely yes”. If the participants answered, “I think so” or “definitely yes”, they were deemed to have the intention to leave.

### 2.5. Assessment of Other Variables

The following variables were considered as covariates and were adjusted for in the analysis: age, gender, educational background (junior high school/high school, vocational school/junior college/technical college, and university/graduate school), employment status (regular or non-regular employment), job description (mainly or not mainly manual work), work frequency (≤3, 4, and ≥5 days/week), and company size (<10, 10–99, 100–999, ≥1000 employees).

### 2.6. Statistical Analysis

All data were expressed as categorical variables using numerical values and percentages. First, a logistic regression analysis involving all participants was performed, with the intention to leave as the dependent variable and the prevalence of cancer as the independent variable. Age, gender, educational background, employment status, job description, work frequency, and company size were used as covariates to adjust for potential confounders. Next, a subsequent logistic regression analysis involving participants with cancer, with the intention to leave as the dependent variable and each health-related problem as the independent variable, was performed. Age, gender, educational background, employment status, job description, work frequency, and company size were used as covariates to adjust for potential confounders. All statistical analyses were performed using Stata version 18.0 (StataCorp LLC, College Station, TX, USA). Statistical significance was set at *p* < 0.05.

## 3. Results

Table 1 presents the participants’ characteristics. The prevalence of cancer was 59.1% (154/4948). The cancer group had a higher proportion of individuals aged >70 years (12.3% vs. 9.5%) and a higher percentage of those with non-regular employment (63.0% vs. 58.2%) than the non-cancer group. Table 2 presents the proportion of participants with intentions to leave their jobs according to their cancer status, along with the adjusted odds ratio (aOR) and 95% confidence interval (95% CI) for the association between cancer and the intention to leave a job. The proportion of participants who intended to leave was higher in the cancer group than in the non-cancer group (33.1% vs. 26.2%). The multivariate logistic regression analysis results indicated that cancer was significantly associated with the intentions to leave (aOR: 1.42, 95% CI: 1.01–2.00, *p* = 0.045).

Table 3 presents the characteristics of the participants with cancer. Of the 154 participants with cancer, 51 (33.1%) intended to leave their jobs. The group with intentions to leave had a higher proportion of workers who worked >5 days weekly than the group without such intentions (70.6% vs. 66.0%). Table 4 presents the prevalence of health-related problems associated with the intention to leave a job and the aOR and 95% CI for the association between each health-related problem and the intention to leave a job. The prevalence of physical (40.7% vs. 24.7%) and mental (62.1% vs. 27.2%) health-related problems was significantly higher in the group with the intention to leave than in the group without this intention. The multivariate logistic regression analysis showed that physical (aOR: 2.33, 95% CI: 1.10–4.92, *p* = 0.026) and mental (aOR: 4.44, 95% CI: 1.80–10.98, *p* = 0.001) health-related problems were significantly associated with the intention to leave. However, problems associated with cancer-related symptoms were not significantly associated with the intention to leave (aOR: 1.01, 95% CI: 0.49–2.11, *p* = 0.969).

## 4. Discussion

The present cross-sectional study revealed that cancer was associated with the intention to leave work among older workers. Furthermore, physical- and mental-health-related issues were associated with the intention to leave among older workers with cancer. These findings suggest the need for physical and mental health support to facilitate the sustained employment of older workers with cancer. The present study is novel because it examined factors associated with the intention to leave among older workers who are survivors of cancer.

The present study found a significant association between cancer and the intention to leave work among older workers. These results are consistent with those of previous studies indicating a higher risk of job loss among survivors of cancer than among the general population [4,5,6], supporting the notion that unemployment among survivors of cancer can lead to serious consequences such as financial toxicity and a decline in the quality of life [7,8,9,10,11,12,13,14]. Medical and occupational health interventions are required to mitigate these negative effects in older workers with cancer.

The present study also highlighted a significant relationship between physical- and mental-health-related issues and the intention to leave among older survivors of cancer. Physical limitations may restrict older workers’ abilities to meet the physical demands of their workplace, and mental-health-related issues may further impair their adaptability and performance. These factors may be associated with a decrease in cancer survivors’ work ability [30,31,32], potentially triggering the intention to leave. Physical- and mental-health-related issues are not exclusive to older survivors of cancer; however, aging may exacerbate these issues. Preventive measures can include multidisciplinary interventions initiated during cancer treatment [33], such as occupational physical exercise, counseling, and psychosocial support at work to facilitate sustained employment.

In contrast, issues associated with cancer-related symptoms were not associated with the intention to leave among older workers who were survivors of cancer. This finding is inconsistent with that of previous studies, namely that cancer-related symptoms are associated with job loss [21,22,23,24,25]. This discrepancy can be attributed to the healthy worker effect, as our survey targeted employed individuals, possibly underestimating the association between symptoms and the intention to leave. In addition, symptoms such as fatigue may have been perceived as physical weakness rather than cancer-related symptoms, potentially leading to an underestimation of their impact on the intention to leave a job. The association between cancer-related symptoms and the intention to leave work was negative in the present study; however, these symptoms have been associated with work outcomes such as loss of productivity [17,30,32,34,35,36,37], emphasizing their importance in the context of sustained employment.

This study has some limitations that warrant further consideration. First, the generalizability of the study findings is limited. We did not consider cancer progression, resulting in the likely underrepresentation of patients with advanced cancer, making the results less applicable to those with severe conditions. This study focused only on tertiary sector workers, so whether these findings apply to those in more physically demanding industries is unclear. Additionally, owing to the recruitment of participants through an internet survey, we could not rule out the possibility that the target population was biased toward relatively well-educated older workers with access to the internet, as well as older adults who are interested in their health. This study targeted only currently employed individuals aged 60–75, who are generally healthier, potentially underestimating the true impact of cancer on employment. Second, this was a cross-sectional study and may not be suitable for drawing causal inferences. However, causal inferences are unlikely to be reversed in the case of the relationship between cancer and intention to leave employment and between physical and mental problems and intention to leave among patients with cancer. In fact, analyses using panel data also suggest that unemployment itself does not affect health, but health problems are associated with unemployment [38]. Third, this study has unmeasured confounding factors. For example, workplaces with good job flexibility and robust occupational health support systems might reduce the impact of cancer and cancer-related health issues on the intention to leave. Conversely, the impact may be more pronounced in workplaces lacking such flexibility and support systems. However, this study does not account for these factors. Future analyses should consider the role of job flexibility and occupational health support systems when examining the effect of cancer and cancer-related health problems on turnover intention. Finally, the results of this study represent the intention to leave, not actual retirement. How many older workers who have the intention to leave the workforce actually leave is unclear, but it is possible that the percentage is small. Therefore, it is not clear how much of an impact a cancer diagnosis or cancer-related health problem has on actual retirement. Future prospective cohort studies are needed to examine the relationship between physical health problems, mental health problems, and cancer-related symptoms and the actual rate of leaving.

## 5. Conclusions

This study elucidated the impact of cancer on the intention to leave work among older workers, highlighting the pivotal role of physical- and mental-health-related issues. The findings suggest that a comprehensive approach to improving physical and mental health is necessary to support the sustained employment of older survivors of cancer. The development of such a comprehensive approach is urgently needed in many developed countries with aging workforces.

## Figures and Tables

**Table 1:** Participants’ characteristics.

	Total (n = 4948)	Cancer	
		Without (n = 4794)	With (n = 154)
Age			
60–64	3348 (67.7%)	3257 (67.9%)	91 (59.1%)
65–69	1127 (22.8%)	1083 (22.6%)	44 (28.6%)
70–75	473 (9.6%)	454 (9.5%)	19 (12.3%)
Gender			
Men	2474 (50.0%)	2399 (50.0%)	75 (48.7%)
Women	2474 (50.0%)	2395 (50.0%)	79 (51.3%)
Educational background			
Junior high school/high school	1432 (28.9%)	1387 (28.9%)	45 (29.2%)
Vocational school/college	1107 (22.4%)	1069 (22.3%)	38 (24.7%)
University	2409 (48.7%)	2338 (48.8%)	71 (46.1%)
Employment status			
Regular employment	2060 (41.6%)	2003 (41.8%)	57 (37.0%)
Non-regular employment	2888 (58.4%)	2791 (58.2%)	97 (63.0%)
Job description			
Not mainly manual work	3374 (68.2%)	3271 (68.2%)	103 (66.9%)
Mainly manual work	1574 (31.8%)	1523 (31.8%)	51 (33.1%)
Work frequency (day/week)			
≤3	889 (18.0%)	862 (18.0%)	27 (17.5%)
4	722 (14.6%)	699 (14.6%)	23 (14.9%)
≥5	3337 (67.4%)	3233 (67.4%)	104 (67.5%)
Company size (employees)			
Micro-scale (<10)	686 (13.9%)	665 (13.9%)	21 (13.6%)
Small-scale (10–49)	1044 (21.1%)	1012 (21.1%)	32 (20.8%)
Medium-scale (50–999)	2018 (40.8%)	1956 (40.8%)	62 (40.3%)
Large-scale (≥1000)	1200 (24.3%)	1161 (24.2%)	39 (25.3%)

**Table 2:** Association between cancer and intention to leave (n = 4948).

	Intention to Leave	Age and Gender Adjusted				Multivariate Adjusted *			
		aOR	95% CI		*p*-Value	aOR	95% CI		*p*-Value
Cancer									
Without (n = 4794)	26.2% (1255/4794)	Reference				Reference			
With (n = 154)	33.1% (51/154)	1.42	1.01	2.00	0.043	1.42	1.01	2.00	0.045

* Adjusted for age, gender, educational background, employment status, job description, work frequency, and company size. aOR, adjusted odds ratio; 95% CI, 95% confidence interval.

**Table 3:** Characteristics of participants with cancer.

	Total (n = 154)	Intention to Leave	
		No (n = 103)	Yes (n = 51)
Age			
60–64	91 (59.1%)	65 (63.1%)	26 (51.0%)
65–69	44 (28.6%)	26 (25.2%)	18 (35.3%)
70–75	19 (12.3%)	12 (11.7%)	7 (13.7%)
Gender			
Men	75 (48.7%)	50 (48.5%)	25 (49.0%)
Women	79 (51.3%)	53 (51.5%)	26 (51.0%)
Educational background			
Junior high school/high school	45 (29.2%)	29 (28.2%)	16 (31.4%)
Vocational school/college	38 (24.7%)	27 (26.2%)	11 (21.6%)
University	71 (46.1%)	47 (45.6%)	24 (47.1%)
Employment status			
Regular employment	57 (37.0%)	39 (37.9%)	18 (35.3%)
Non-regular employment	97 (63.0%)	64 (62.1%)	33 (64.7%)
Job description			
Not mainly manual work	103 (66.9%)	68 (66.0%)	35 (68.6%)
Mainly manual work	51 (33.1%)	35 (34.0%)	16 (31.4%)
Work frequency (day/week)			
≤3	27 (17.5%)	19 (18.4%)	8 (15.7%)
4	23 (14.9%)	16 (15.5%)	7 (13.7%)
≥5	104 (67.5%)	68 (66.0%)	36 (70.6%)
Company size (employees)			
Micro-scale (<10)	21 (13.6%)	12 (11.7%)	9 (17.6%)
Small-scale (10–49)	32 (20.8%)	25 (24.3%)	7 (13.7%)
Medium-scale (50–999)	62 (40.3%)	40 (38.8%)	22 (43.1%)
Large-scale (≥1000)	39 (25.3%)	26 (25.2%)	13 (25.5%)

**Table 4:** Association of physical-health-related issues, mental-health-related issues, and cancer-related symptoms with the intention to leave (n = 154).

	Intention to Leave	Age-Gender Adjusted				Multivariate Adjusted *			
		aOR	95% CI		*p*-Value	aOR	95% CI		*p*-Value
Physical-health-related issue									
Without (n = 73)	24.7% (18/73)	Reference				Reference			
With (n = 81)	40.7% (33/81)	2.09	1.04	4.23	0.039	2.33	1.10	4.92	0.026
Mental-health-related issue									
Without (n = 125)	27.2% (34/125)	Reference				Reference			
With (n = 29)	62.1% (18/29)	3.69	1.59	8.58	0.002	4.44	1.80	10.98	0.001
Cancer-related symptoms									
Without (n = 99)	33.3% (33/99)	Reference				Reference			
With (n = 55)	32.7% (18/55)	1.03	0.51	2.09	0.937	1.01	0.49	2.11	0.969

* Adjusted for age, gender, educational background, employment status, job description, work frequency, and company size. aOR, odds ratio; 95% CI, 95% confidence interval.

## Data Availability

The original contributions presented in the study are included in the article, further inquiries can be directed to the corresponding author.
